# Stress-tolerance genes in rice: bridging gene discovery, functional validation, and breeding applications

**DOI:** 10.3389/fpls.2026.1835666

**Published:** 2026-06-08

**Authors:** Mvuyeni Nyasulu, Rudoviko Galileya Medison, Waqas Mushtaq, Stanley Nyenje Mataka

**Affiliations:** 1 Horticultural Department, Lilongwe University of Agriculture and Natural Resources (LUANAR), Lilongwe, Malawi; 2 Key Laboratory of South China Agricultural Plant Molecular Analysis and Genetic Improvement, South China Botanical Garden, Chinese Academy of Sciences, Guangzhou, China; 3 Jiangxi Agricultural University, Key Laboratory of Crop Physiology, Ecology, and Genetic Breeding, Ministry of Education/College of Agronomy, Nanchang, China; 4 College of Agriculture, Yangtze University, Jingzhou, China

**Keywords:** drought stress, functional characterization, genome editing, genomic selection, GWAS, marker-assisted selection, QTL mapping, rice

## Abstract

Rice productivity is increasingly threatened by abiotic stresses, with drought being a major constraint to stable yields under changing climate conditions. Advances in genomics and high-throughput omics technologies have identified numerous drought-responsive genes in rice through transcriptomics, genome-wide association studies (GWAS), and quantitative trait locus (QTL) mapping. While these approaches have improved our understanding of the genetic basis of drought responses, the practical application of gene discovery in breeding programs remains limited. This review focuses on functionally validated drought-tolerance genes, emphasizing candidates confirmed through reverse genetics approaches, such as gene knockout, overexpression, and genome editing. Key examples are discussed across major functional categories, including transcriptional regulation, signal transduction, and cellular protection mechanisms, highlighting their roles in drought tolerance and, when relevant, yield-related traits. We also explore how validated drought-tolerance genes have been integrated into rice improvement strategies, including marker-assisted selection, genomic selection, and genome editing. Major challenges are addressed, such as inadequate evaluation under combined stress conditions and weak connections between gene function and field performance. By combining evidence from genomics, functional biology, and breeding research, this review outlines priority research directions to accelerate the translation of candidate genes into climate-resilient rice cultivars. Rather than providing a comprehensive list of drought-responsive genes, this review emphasizes high-confidence, functionally characterized targets with practical relevance for breeding.

## 1 Introduction

Rice is a staple food for more than half of the global population and is central to food security, particularly in developing countries (Darko et al., 2025). However, rice production is increasingly threatened by climate change, with drought emerging as one of the most significant constraints to stable yields (Li et al., 2024). A large proportion of rice is cultivated under rainfed conditions, where water availability is often unpredictable, making crops highly vulnerable to drought stress (Zampieri et al., 2023). Drought affects rice growth and productivity at multiple developmental stages, including seedling establishment, vegetative growth, and grain filling (Sadhukhan et al., 2024). At the physiological level, drought stress limits water uptake, reduces photosynthetic efficiency, and induces oxidative damage, ultimately leading to yield loss (Bijalwan et al., 2025; Song et al., 2026). These responses are regulated by complex genetic networks, highlighting the quantitative and multifaceted nature of drought tolerance in rice.

Advances in genomics and high-throughput technologies have accelerated the identification of drought-responsive genes through approaches such as transcriptomics, genome-wide association studies (GWAS), and quantitative trait locus (QTL) mapping (Mir et al., 2012; Sahoo et al., 2023; Wang et al., 2023; Zhao et al., 2023; Jin et al., 2025) (Zhang, et al., 2022). These studies have provided valuable insights into the genetic basis of drought tolerance. However, despite the large number of candidate genes identified, their practical application in breeding programs remains limited.

A key limitation lies in the gap between gene discovery and functional validation. While many genes have been associated with drought response, only a small proportion have been experimentally validated using reverse genetics approaches, such as gene knockout, overexpression, and genome editing. Functional validation is essential to establish causal relationships between genes and phenotypes, particularly when evaluated under realistic field-like stress conditions that capture the complexity and variability of natural environments (Šimon et al., 2025).

Furthermore, the integration of validated genes into breeding programs is constrained by factors such as genotype-by-environment interactions, limited evaluation under combined stress conditions, and weak links between genes function and field performance (Crossa, 2012; Wang et al., 2025). These challenges have slowed the translation of genomic discoveries into improved drought-tolerant rice varieties.

This review addresses these limitations by focusing on functionally validated drought-tolerance genes in rice. We emphasize genes confirmed through reverse genetics approaches and categorize them based on their biological functions, including transcriptional regulation, signal transduction, and cellular protection mechanisms. In addition, we discuss how these genes can be effectively integrated into modern breeding strategies, such as marker-assisted selection, genomic selection, and genome editing. By bridging the gap between gene discovery and application, this review aims to provide insights into accelerating the development of drought-resilient rice cultivars.

## 2 Genetic basis of drought tolerance in rice

Understanding how rice responds to drought at the genetic level is crucial for developing resilient varieties that can withstand water-limited conditions. Drought tolerance is not the result of a single gene or pathway; rather, it is a complex quantitative trait controlled by multiple genes distributed across the genome, each contributing small to moderate effects to the overall phenotype (Bashir et al., 2021; Haghpanah et al., 2024). The challenge is that drought affects multiple physiological processes and developmental stages, making the plant’s response highly context-dependent and influenced by environmental conditions (Rehaman et al., 2025).

### 2.1 Complexity of drought tolerance

**Figure 1:**
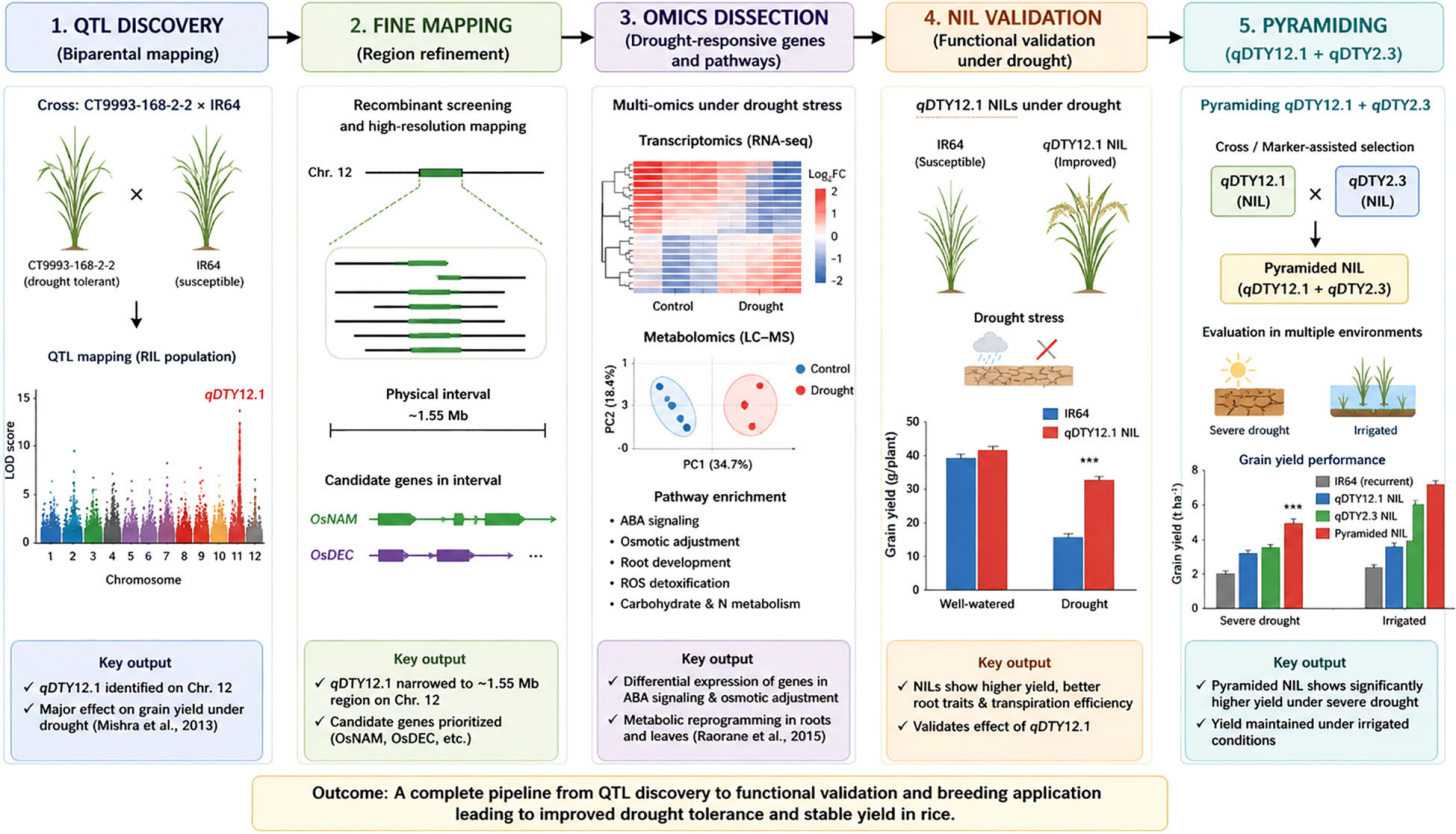
Multilevel complexity of drought tolerance in rice.

Drought tolerance is the result of many interconnected plant responses, working from the molecular to the whole-plant level (Haghpanah et al., 2024) (**Figure 1**). At the molecular level, drought triggers stress signaling networks involving transcription factors (Hu et al., 2022), kinases (Chen et al., 2021; Ali et al., 2025) and hormone pathways such as abscisic acid (ABA) (Muhammad Aslam et al., 2022; Kim et al., 2024). At the cellular level, mechanisms like osmolyte accumulation, reactive oxygen species scavenging, and membrane stabilization help maintain cellular function (Hussain et al., 2019; Ghosh et al., 2021; Guru et al., 2026). Physiologically, traits like root growth (Gupta et al., 2020; Zhang et al., 2025) stomatal regulation (Luo et al., 2019; Rhaman et al., 2025) and photosynthesis (Chauhan et al., 2023) all contribute to the plant’s ability to cope with water deficit.

Because drought tolerance is polygenic, each gene usually has a small effect, and the way genes interact with each other and with the environment can greatly influence the plant’s performance (Shelake et al., 2022; Liu et al., 2023). For example, a gene that promotes deep root growth may be highly beneficial in one environment but less so under combined drought and heat stress. This complexity makes it necessary to use advanced genomic tools and statistical models to understand the trait fully.

### 2.2 Traits involved in drought tolerance

Rice survives drought through a combination of morphological, physiological, and biochemical traits that work together to determine its overall resilience. Root architecture is one of the most important traits for drought avoidance, as deeper and more extensive roots allow plants to access water from deeper soil layers. This ability is closely linked to the DEEPER ROOTING 1 (*DRO1*) gene, which controls root growth angle and promotes deeper rooting (Guseman et al., 2017). Other genes, such as *OsWOX11* (Zhao et al., 2009) and *OsNAC5* (Jeong et al., 2013), also contribute to root development and help plants adjust their root systems under water-limited conditions.

Stomatal regulation and transpiration efficiency help plants conserve water while still maintaining photosynthesis. These processes are largely controlled by abscisic acid (ABA) signaling. Gene such as *OsSRO1c* play key roles in regulating stomatal closure, reducing water loss during drought (You et al., 2013). In addition, transcription factors like *OsbZIP23* (Xiang et al., 2008) and *OsDREB2A* (Zhang et al., 2013) coordinate the expression of stress-responsive genes, allowing the plant to balance water conservation with carbon assimilation.

At the cellular level, osmotic adjustment helps maintain cell turgor and protects cellular functions during water deficit. This is achieved through the accumulation of solutes such as proline and sugars, which are regulated by genes like *OsP5CS* (Hur et al., 2004) and *OsTPS1* (Li et al., 2011). At the same time, drought stress often leads to the production of reactive oxygen species (ROS), which can damage cells. To counter this, antioxidant enzymes encoded by genes such as *OsCSD1*, *OsCSD4*, and *OsMSD1* help detoxify ROS and maintain cellular stability (Shiraya et al., 2015; Sanyal et al., 2022).

The stay-green trait and delayed leaf senescence allow plants to maintain photosynthetic activity for longer periods under drought, supporting grain filling and yield stability. These traits are associated with genes such as *OsSGR* and *NYC1*, which regulate chlorophyll degradation and help preserve leaf function. Genes like *OsRbcS* further support continued photosynthesis under stress conditions (Thomas and Ougham, 2014; Ramkumar et al., 2019; Bheemanahalli et al., 2021).

Visible adaptations such as leaf rolling and reduced canopy temperature also play a role in reducing water loss. Leaf rolling, for example, is linked to genes like *OsZHD1* and *OsRPK1*, which influence leaf structure and bulliform cell function. Canopy temperature, often used as an indicator of plant water status, reflects the plant’s ability to regulate transpiration efficiently (Kadioglu et al., 2012; Ali et al., 2022; Zhou et al., 2023).

Overall, these traits are controlled by complex, interacting genetic networks rather than single genes. Major drought-related QTLs, such as *qDTY1.1*, *qDTY3.1*, and *qDTY12.1*, bring together multiple traits, including root architecture, water-use efficiency, and yield stability under stress. The coordinated action of regulatory genes, signaling pathways, and physiological processes ultimately determines how well rice plants tolerate drought. Understanding these links between genes and traits is essential for guiding functional studies and developing improved varieties through modern breeding approaches (Passioura, 2012; Hassan et al., 2023).

### 2.3 Genetic dissection of drought tolerance: QTLs, GWAS, and transcriptomic insights

Drought tolerance in rice has been extensively dissected through quantitative trait loci (QTL) mapping, leading to the identification of several major loci associated with grain yield and adaptive responses under water deficit conditions. Notable examples include *qDTY1.1, qDTY3.1, qDTY6.1*, and *qDTY12.1*, which consistently influence grain yield under reproductive-stage drought across diverse genetic backgrounds and environments (**Table 1**) (Vikram et al., 2011a; Mishra et al., 2013; Dixit et al., 2014). Among these, *qDTY12.1* has been shown to explain a substantial proportion of phenotypic variation in yield under drought, while also maintaining performance under non-stress conditions, making it highly valuable for breeding programs (Mishra et al., 2013). Similarly, *qDTY3.1*, identified in the Swarna/Apo population, enhances drought adaptation and shows epistatic interactions with other qDTY loci, improving overall performance under stress (Solis et al., 2018). Meta-analysis of multiple QTL studies has further refined these regions into stable meta-QTLs, increasing their reliability for deployment in breeding pipelines (Swamy et al., 2011).

**Table 1:** Major drought-related quantitative trait loci (QTLs) identified in rice.

QTL name	Trait/effect	Chromosome	Genetic background	Reference
*qDTY12.1*	Grain yield under reproductive-stage drought	12	Consistent effect across environments; high phenotypic variance explained (~23–51%)	(Mishra et al., 2013)
*qDTY1.1*	Grain yield under reproductive-stage drought (large and consistent effect)	1	Identified in N22 × Swarna/IR64/MTU1010 backgrounds	(Vikram et al., 2011a)
*qDTY3.1*	Grain yield under drought; interacts with other qDTYs to enhance drought tolerance	3	Identified in Swarna/Apo population	(Solis et al., 2018)
*qDTY2.3*	Grain yield under severe drought (enhances effect of qDTY12.1)	2	Works synergistically with qDTY12.1	(Solis et al., 2018)
*qDTY3.2*	Drought recovery traits (canopy temperature, shoot weight)	3	Affects biomass and recovery after stress	(Dixit et al., 2014)
*qDTY6.1*	Grain yield under drought; consistent across seasons	6	Identified in backcross populations (e.g., TDK1 × IR55419-04)	(Dixit et al., 2014)
*qDTY6.2*	Drought/Irrigation response	6	Affects grain yield under stress and non-stress	(Dixit et al., 2014)

**Figure 2:**
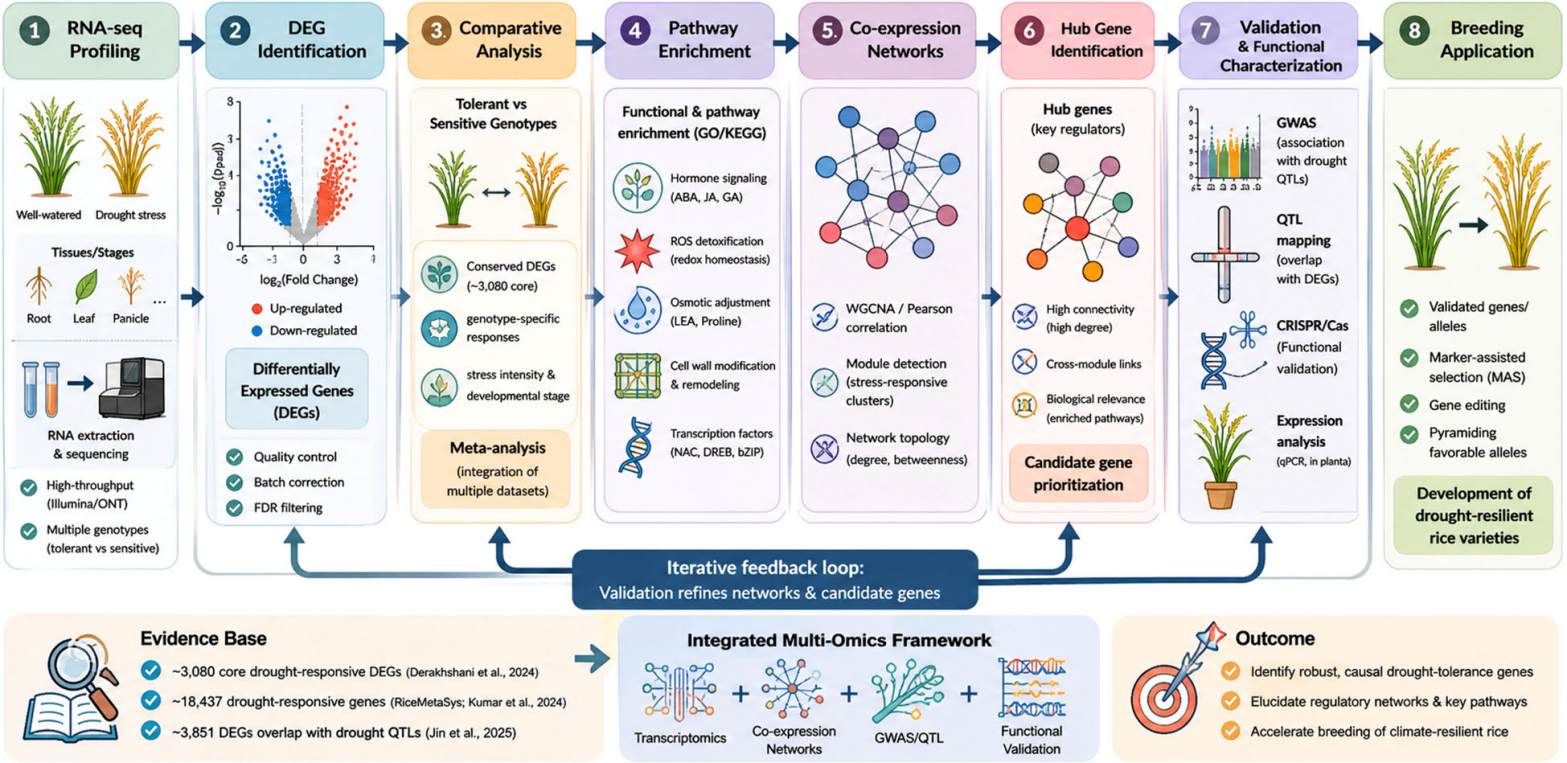
Integrated genetic dissection and breeding pipeline for drought tolerance in rice.

A representative example of a worked genetic dissection pipeline is the pyramiding and refinement of *qDTY12.1* and *qDTY2.3*. Initially identified through biparental QTL mapping, *qDTY12.1* was subsequently validated across multiple genetic backgrounds for its strong and stable effect on grain yield under drought stress (Vikram et al., 2011b; Mishra et al., 2013; Tseng et al., 2025). Fine-mapping and multi-environment evaluation narrowed the locus to a major-effect genomic region on chromosome 12, establishing its consistent contribution to drought yield performance. Complementary transcriptomic and metabolomic analyses of near-isogenic lines revealed drought-responsive regulation of genes involved in ABA signaling, osmotic adjustment, and root system modulation, highlighting key physiological pathways underlying the QTL effect (Raorane et al., 2015). These integrative omics approaches further supported prioritization of stress-responsive candidate genes within the region, although functional validation remains essential for causal gene identification. When pyramided with *qDTY2.3*, which enhances drought response stability across environments, the resulting near-isogenic lines exhibited significantly improved grain yield under severe drought while maintaining performance under irrigated conditions (Shamsudin et al., 2016). This demonstrates a complete translational pipeline from QTL discovery through molecular dissection to breeding application for drought-tolerant rice improvement (**Figure 2**).

Genome-wide association studies (GWAS) complement QTL mapping by examining natural genetic variation across diverse rice populations (Desaint et al., 2023; He and Gai, 2023). GWAS links single nucleotide polymorphisms (SNPs) to drought-responsive traits with higher resolution, identifying candidate genes that influence root system architecture, osmotic regulation, and developmental timing under stress (Siangliw et al., 2022; Wei et al., 2023; Yi et al., 2023; Ghazy et al., 2024). By combining GWAS and QTL data, researchers can refine genomic regions of interest and focus on genes most likely to contribute to drought resilience.

Transcriptomic studies, particularly RNA sequencing (RNA-seq), add another layer of insight by showing which genes are actively responding to drought (Raorane et al., 2015). Comparing tolerant and sensitive rice varieties under controlled or field conditions reveals stress-responsive pathways and regulatory genes, providing a functional connection between DNA regions identified by QTL or GWAS and the plant’s actual response to drought (Chengqi et al., 2024). Integrating RNA-seq with mapping studies helps prioritize candidates for further testing through knockout, overexpression, or genome-editing experiments.

Despite these advances, translating candidate loci into functionally validated genes remains a challenge. Many regions identified by QTLs or GWAS contain numerous genes, but only a small fraction have been experimentally confirmed. An integrative approach combining mapping, transcriptomics, and functional validation allows breeders to stack beneficial alleles, apply genomic selection, and use gene editing to develop rice varieties that maintain high yields even under drought conditions.

## 3 Identification of drought-responsive genes

### 3.1 Transcriptomics

**Figure 3:**
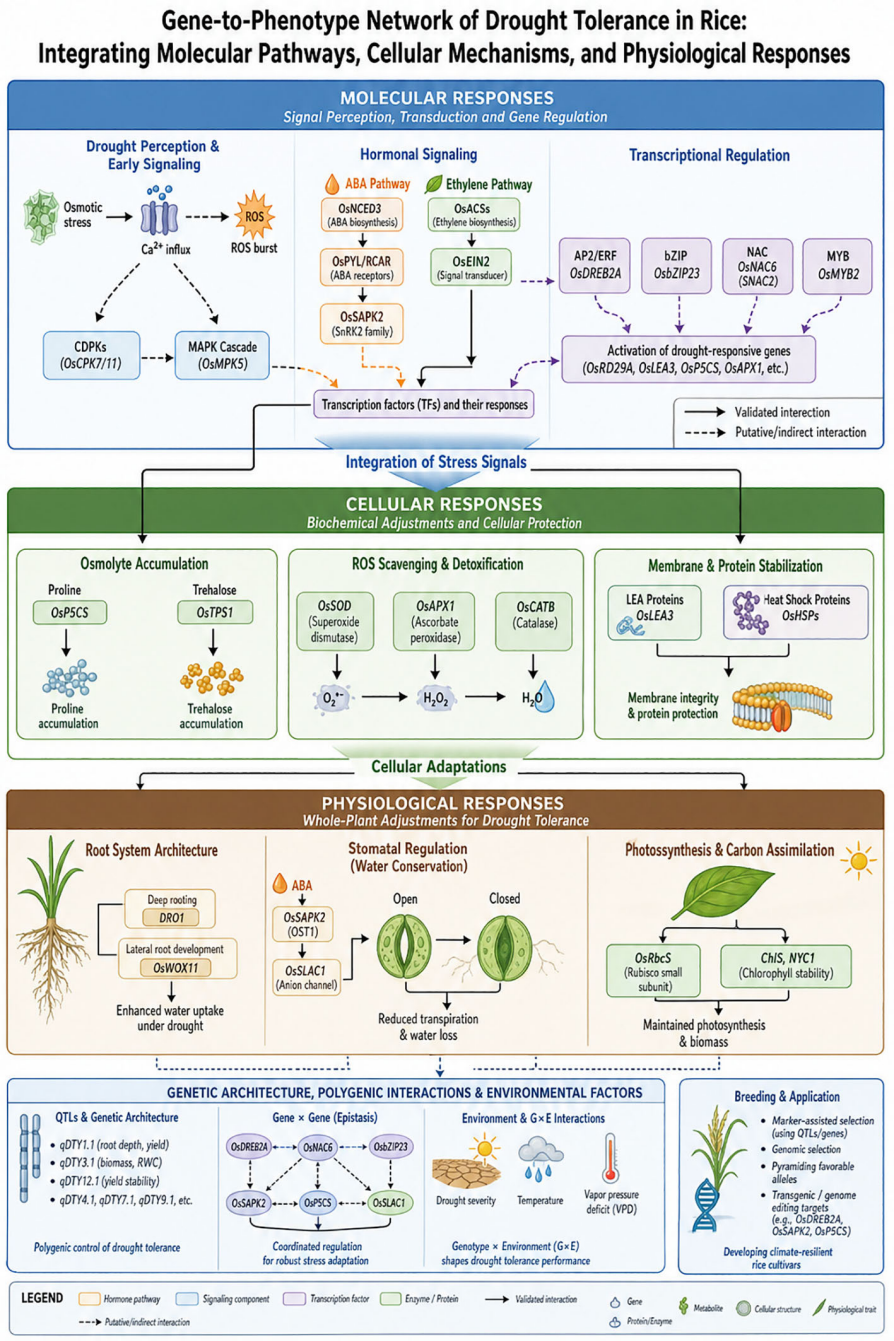
Transcriptomic dissection of stress tolerance in rice: from RNA-seq to gene networks and breeding application.

Transcriptomics has become a central tool for dissecting drought tolerance in rice, enabling genome-wide analysis of gene expression changes underlying stress adaptation. The field has progressed from low-throughput hybridization-based methods to RNA sequencing technologies that provide high-resolution, whole-transcriptome profiling across tissues and environmental conditions (**Figure 3**). This transition has enabled a detailed reconstruction of gene regulatory networks involved in drought response and genotype-specific adaptation.

A significant conceptual advancement in transcriptomic studies is the understanding that changes in gene expression induced by drought reflect both adaptive regulatory responses and secondary effects of cellular damage. As a result, only a subset of differentially expressed genes (DEGs) is directly linked to drought tolerance, highlighting the need for comparative and integrative approaches to identify functionally relevant genes.

Comparative transcriptomics, which involves comparing drought-tolerant and drought-sensitive genotypes, is a valuable strategy for identifying stress-responsive genes. This approach utilizes natural genetic variation in rice to prioritize differentially expressed genes (DEGs) involved in adaptive responses. These DEGs can then be further studied for functional validation, allele mining, and molecular breeding purposes.

Large-scale analyses have revealed both conserved and genotype-specific transcriptional responses to drought. Meta-analyses have identified approximately 3,080 core drought-responsive DEGs consistently regulated across tolerant genotypes, suggesting the existence of a conserved transcriptional backbone underlying drought adaptation (Derakhshani et al., 2024). Additionally, integrative resources such as RiceMetaSys have compiled around 18,437 drought-responsive genes from diverse RNA-seq datasets, reflecting extensive transcriptional plasticity across rice genotypes (Kumar et al., 2024). Moreover, combined transcriptomic and meta-QTL analyses have identified approximately 3,851 DEGs overlapping with drought-associated genomic regions, strengthening the link between transcriptional regulation and genetic loci controlling drought tolerance (Jin et al., 2025).

Collectively, these findings demonstrate that drought stress triggers highly complex and multilayered transcriptional networks rather than a single linear pathway. Across studies, drought tolerance is consistently associated with regulation of hormone signaling (ABA, jasmonate, and gibberellin pathways), transcription factors, ROS detoxification systems, osmotic adjustment mechanisms, and cell wall modification processes. Network-based analyses further identify hub genes regulating stress signaling and redox homeostasis, highlighting the modular and systems-level nature of drought adaptation.

Despite substantial progress, variability in genotypes, stress protocols, developmental stages, tissues, and analytical pipelines limits direct comparison across studies. Therefore, integrative frameworks combining transcriptomics with QTL mapping, co-expression networks, and functional validation are essential to identify robust and biologically meaningful candidate genes for drought-resilient rice improvement.

### 3.2 Genetic mapping (QTL/GWAS)

Genome-wide association studies (GWAS) and quantitative trait locus (QTL) mapping have been central to dissecting the complex genetic architecture of drought tolerance in rice. These approaches enable the identification of genomic regions controlling key adaptive traits such as yield stability, root architecture, stomatal regulation, and physiological water-use efficiency under water-limited conditions.

Classical linkage-based QTL mapping has identified numerous drought-responsive loci across diverse rice populations. Meta-analyses integrating multiple independent QTL studies have compiled approximately 653 drought-related QTLs, which were subsequently refined into around 70 meta-QTL regions with improved resolution and stability across environments (Selamat and Nadarajah, 2021). These meta-QTLs provide more robust genomic targets by reducing redundancy and narrowing confidence intervals, thereby facilitating candidate gene identification.

With the advent of high-throughput genotyping, GWAS has enabled finer dissection of natural allelic variation associated with drought response. High-density GWAS in diverse rice panels have identified stable SNP-trait associations linked to grain yield, biomass, and physiological drought tolerance traits, including 26 robust loci under water-deficit conditions (Ghazy et al., 2024). Similarly, studies in rice landraces have revealed multiple drought-associated QTLs governing root system architecture, osmotic adjustment, and seedling-stage survival, highlighting the importance of local adaptation and genetic diversity (Hoang et al., 2019).

Importantly, many of these mapped loci co-localize with genes involved in key biological pathways, including abscisic acid (ABA) signaling, root development regulation, osmoprotectant biosynthesis, and reactive oxygen species (ROS) detoxification. This has enabled the prioritization of candidate genes such as transcription factors, protein kinases, and transporters that regulate drought perception and downstream stress responses.

Despite these advances, a major challenge remains the large number of candidate genes within individual QTL regions, often limiting direct functional interpretation. To address this limitation, integrative approaches combining GWAS/QTL mapping with transcriptomics, haplotype analysis, and functional genomics tools are increasingly being used to refine candidate gene lists and establish causality.

Collectively, QTL and GWAS studies demonstrate that drought tolerance in rice is a highly polygenic trait governed by a complex network of interacting genomic regions. These findings provide a foundational resource for marker-assisted selection, genomic selection, and gene editing strategies aimed at improving drought resilience in rice breeding programs.

## 4 Functional validation of drought tolerance genes

Although transcriptomic and genetic mapping studies have identified thousands of drought-responsive genes in rice, only a limited number have been functionally validated through genetic manipulation or phenotypic assessment. As reported by the National Rice Data Center (https://www.ricedata.cn/), 262 drought tolerance–related genes had been cloned as of 2020. Since then, numerous additional genes have been identified and functionally characterized in independent studies.

Recent studies have identified specific drought-responsive genes such as *DROT1, OsSPL10*, and *OsERF103*, but their functional characterization varies significantly in depth and physiological context (Wang et al., 2025). For example, *DROT1*, a COBRA-like cell wall protein, has been validated through loss-of-function and overexpression experiments under controlled soil drought and field drought conditions (Sun et al., 2022). It consistently improved seedling survival rate, biomass retention, and plant height maintenance during prolonged water deficit. Mechanistically, *DROT1* enhances drought tolerance by altering cell wall architecture, increasing cellulose deposition, and maintaining cellulose crystallinity. This strengthens vascular tissues and stabilizes plant water transport. Its function is regulated by *ERF3* (a negative regulator) and *ERF71* (a positive regulator), connecting drought signaling directly to cell wall remodeling. Additionally, beneficial haplotypes of *DROT1* were linked to enhanced performance in upland rice cultivated under aerobic field conditions.

Similarly, *OsSPL10*, a member of the SBP-box transcription factor family, contributes to drought adaptation by regulating reactive oxygen species (ROS) homeostasis and stomatal behavior (Li et al., 2023). This modulation influences transpiration efficiency and water loss control under drought stress. However, most evidence supporting this comes from greenhouse and controlled stress assays rather than long-term field drought validation. On the other hand, *OsERF103*, an AP2/ERF transcription factor, plays a role in ethylene-responsive signaling networks and has been linked to drought response by regulating downstream stress-inducible genes (Yang et al., 2024). Its effects are mainly observed at the seedling stage under PEG-induced osmotic stress or short-term soil drying, with limited evidence from field-scale drought environments.

Collectively, these examples illustrate that while core drought-responsive genes have been identified, their functional validation is highly variable in terms of mechanistic depth and experimental robustness. Recent syntheses confirm that only a small fraction of drought-associated loci identified through transcriptomic and genetic mapping approaches have been experimentally validated under field-relevant conditions (Giri et al., 2021; Gong et al., 2025). For instance, targeted phenotypic analyses in diverse rice germplasm validated functional roles for only about 16 out of dozens of candidate genes, illustrating the substantial gap between gene discovery and experimental validation (Singh et al., 2024).

Functionally validated drought-tolerance genes include transcription factors (e.g., NAC, DREB, bZIP families), signaling components (protein kinases, ABA receptors), and stress-protection genes (ROS scavengers, osmoprotectant regulators) (Todaka et al., 2015; Yang et al., 2022; Bhattacharjee et al., 2023). These genes act at multiple levels to enhance drought resilience, from regulating gene expression and signaling cascades to maintaining cellular homeostasis and root system architecture (Todaka et al., 2015; Rasheed et al., 2020). A complete list of some validated genes, their functional roles, and observed drought phenotypes is provided in **Supplementary Table 1**.

**Figure 4:**
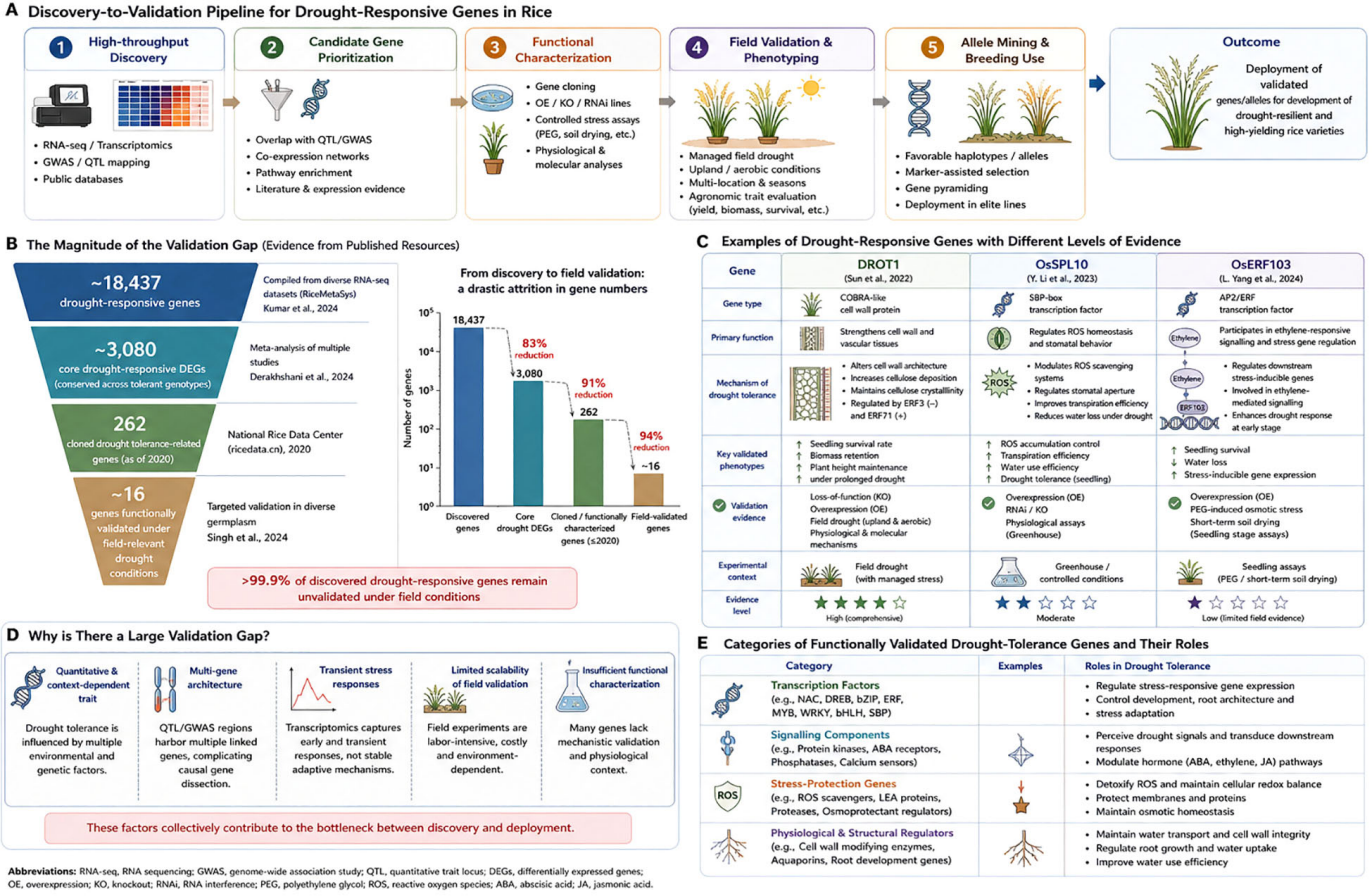
Schematic overview of the pipeline from gene discovery to functional validation in rice under drought stress. **(A)** Discovery-to-validation pipeline illustrating high-throughput discovery, candidate gene prioritization, functional characterization, field validation and phenotyping, allele mining, and breeding application. **(B)** The magnitude of the validation gap between discovered drought-responsive genes and those validated under field drought conditions. **(C)** Examples of drought-responsive genes with varying levels of experimental validation and evidence. **(D)** Key factors contributing to the large validation gap in drought-related gene studies. **(E)** Categories of functionally validated drought-tolerance genes and their roles in drought adaptation and stress tolerance in rice.

Overall, the discrepancy between gene discovery and functional validation reflects the quantitative and context-dependent nature of drought tolerance, the multi-gene architecture of the trait, and the limited scalability of field-based validation experiments. While transcriptomic studies often capture transient stress responses, QTL and GWAS regions typically encompass multiple linked genes, complicating causal gene identification. A conceptual overview of this discovery-to-validation pipeline is illustrated in **Figure 4**, highlighting the need for integrative functional genomics approaches to bridge the gap between candidate gene discovery and deployment in drought-resilient rice breeding.

### 4.1 Functional classification of validated genes

Rice drought tolerance is regulated by an interconnected gene regulatory network involving transcriptional control, signal perception and transduction, and cellular protection mechanisms. Rather than acting independently, these components form hierarchical pathways integrating hormone signaling (particularly ABA), calcium and ROS dynamics, and developmental reprogramming to coordinate whole-plant adaptation to water deficit (Ma et al., 2024).

#### 4.1.1 Transcription factors (regulatory hierarchy and gene expression control)

Transcription factors function as central regulators that integrate upstream hormonal and stress signals into coordinated transcriptional reprogramming. Many drought-responsive transcription factors operate through ABA-dependent and ABA-independent pathways, binding to cis-regulatory elements in promoters to activate stress-responsive gene networks (Hussain et al., 2021).

For example, bZIP family members such as *OsbZIP23, OsbZIP46, OsbZIP62*, and *OsbZIP12* regulate drought tolerance primarily through ABA-responsive element (ABRE)-mediated transcriptional activation. These factors bind promoter regions of downstream stress-inducible genes involved in osmoprotection, antioxidant defense, and stomatal regulation, thereby linking ABA perception to transcriptional output (Xiang et al., 2008; Tang et al., 2012; Joo et al., 2014; Yang et al., 2019). These regulators therefore represent key ABA-responsive transcriptional hubs that coordinate physiological drought responses (**Supplementary Table 1**).

In contrast, *OsDREB1A* functions mainly through an ABA-independent pathway by binding dehydration-responsive elements (DRE/CRT motifs), enabling rapid activation of stress-responsive genes involved in osmolyte biosynthesis and protective metabolic pathways (Dubouzet et al., 2003). This pathway is particularly important during early stress perception, when ABA accumulation is still limited.

Similarly, *OsNAC14* integrates developmental and stress-related signals by regulating genes associated with cell wall modification and cellular stabilization. Through transcriptional control of structural and protective genes, it contributes to maintaining cellular integrity under dehydration stress (Shim et al., 2018). Collectively, these transcription factors operate in a hierarchical regulatory framework, where bZIP and NAC proteins function as higher-order regulatory hubs, while DREB factors mediate rapid early stress responses.

#### 4.1.2 Signaling pathways (signal perception, transduction, and hormonal integration)

Drought perception in rice is mediated through receptor and kinase-mediated signaling cascades that convert environmental water deficit into intracellular biochemical signals.

ABA receptors such as *OsPYL* proteins initiate the canonical ABA signaling pathway by binding ABA and inhibiting *PP2C* phosphatases, thereby activating *SnRK2* kinases. This phosphorylation cascade regulates downstream transcription factors and ultimately induces stomatal closure to reduce transpirational water loss (Bhatnagar et al., 2020; Hsu et al., 2021). This system forms a core ABA-dependent drought signaling module.

Auxin-responsive genes such as *OsIAA6* and *OsIAA20* further contribute by modulating growth regulation under drought stress. Through interaction with auxin response factors (ARFs), these proteins adjust gene expression programs controlling cell elongation and developmental plasticity, thereby reducing growth demand under stress conditions (Jung et al., 2015; Zhang et al., 2021).

At a broader level, receptor-like kinases and secondary messengers such as calcium ions (Ca²^+^) and reactive oxygen species (ROS) act as early stress signals, forming interconnected signaling networks that amplify drought perception. Calcium spikes activate downstream kinases, while ROS functions both as a damaging agent and signaling molecule, enabling feedback regulation between stress perception and response (Memon and Duraković, 2015; Zhang et al., 2018).

#### 4.1.3 Cellular protection mechanisms (physiological and structural effectors)

Effector genes execute the final physiological adaptations required for drought survival by stabilizing cellular structures and modifying plant architecture.

*OsLEA3-1*, a late embryogenesis abundant (LEA) protein, protects cellular proteins and membranes through molecular shielding and water replacement functions, thereby maintaining cellular integrity under dehydration stress (Hu et al., 2014). This represents a key component of osmotic stress tolerance.

Root architecture is strongly influenced by genes such as *DRO1*, which regulates gravitropic root growth and promotes deeper rooting angles, enhancing water acquisition from deeper soil layers (Uga et al., 2015; Guseman et al., 2017). This represents a major morphological adaptation contributing to drought avoidance.

Similarly, *OsRCI2–5* maintains membrane stability under dehydration by regulating membrane-associated stress responses and lipid homeostasis, thereby preserving cellular transport and metabolic function during water deficit (Li et al., 2014).

#### 4.1.4 Integrated network perspective and breeding implications

Collectively, drought tolerance in rice is governed by a multilayered regulatory hierarchy comprising transcriptional regulators, hormone- and kinase-mediated signaling pathways, and structural effector genes. These components are interconnected through ABA-dependent and independent networks, calcium and ROS crosstalk, and feedback regulation between gene expression and physiological adaptation.

Although over 200 drought-responsive genes have been cloned in rice, functional validation indicates that most act within specific network modules rather than isolated pathways. Overexpression and knockout studies consistently demonstrate that manipulating key regulatory nodes can significantly enhance drought tolerance (Li and Wang, 2016; Verma et al., 2019; Long et al., 2023; Yan et al., 2023). However, many genes have been characterized primarily under controlled conditions, and their network-level interactions under field environments remain insufficiently resolved.

Future efforts should therefore focus on systems-level integration of transcriptional networks, signaling cascades, and physiological traits, combined with multi-omics and field-based validation, to identify robust genetic modules for drought-resilient rice breeding.

## 5 Future directions and prioritization strategies for functional validation

The large number of drought-responsive genes identified in rice presents both an opportunity and a challenge. While these studies have generated a wealth of potential candidates, only a small fraction can realistically be tested through functional validation. This makes it essential to move beyond broad discovery and focus on identifying the most promising genes. In practice, this means using smarter, more targeted approaches that can narrow down candidates and improve the chances of identifying genes that truly contribute to drought tolerance.

One way forward is through the integration of multi-omics data, where transcriptomic information is combined with genomic, proteomic, and metabolomics datasets (Arriagada et al., 2025; Dakal et al., 2025; Jin et al., 2025; Naing et al., 2025). When the same gene appears across different types of data, it is more likely to play a meaningful role in drought response. For instance, genes that are both differentially expressed under drought and located within QTL or GWAS regions provide stronger evidence for functional relevance. This kind of integration helps reduce noise and allows researchers to focus on candidates with greater biological significance.

Another useful approach is network-based analysis, which looks at how genes interact rather than considering them in isolation. Co-expression and regulatory networks can highlight key “hub” genes that control multiple downstream processes. Targeting these genes may have a broader and more consistent impact on drought tolerance, making them attractive candidates for validation.

At the same time, advances in high-throughput reverse genetics are making it easier to test gene function more efficiently. Technologies such as CRISPR/Cas systems, RNA interference, and mutant libraries now allow researchers to study multiple genes in parallel (Ben-Amar et al., 2016; Liu et al., 2020; Wang et al., 2022). When combined with improved phenotyping tools, these approaches can significantly speed up the validation process and reduce the time required to link genes to drought-related traits.

Equally important is the need for field-relevant phenotyping. Many genes that perform well under controlled conditions do not always translate into improved performance in real farming environments (Baslam et al., 2023; Kim et al., 2026). Testing candidate genes under realistic drought scenarios across different growth stages and environmental conditions is essential to ensure that validated genes have practical value.

Overall, bridging the gap between gene discovery and functional validation will require a more focused and integrated approach. By combining multi-omics data, network-based prioritization, high-throughput validation tools, and field-based testing, researchers can better identify genes that genuinely contribute to drought tolerance and support the development of more resilient rice varieties.

## 6 Conclusion

In the last decade, advancements in transcriptomics, QTL mapping, and genome-wide association studies have expanded the repertoire of drought-responsive genes in rice. However, functional validation has shown that only a subset consistently contributes to drought tolerance across different environments. Current evidence suggests that the most effective mechanisms are concentrated within key regulatory modules rather than spread across all identified candidates. Specifically, ABA-dependent transcription factors, particularly bZIP and NAC family members, serve as central hubs controlling downstream responses like osmotic adjustment, ROS detoxification, and stomatal regulation. The core ABA signaling pathways (*PYL–PP2C–SnRK2*) form the primary framework for perceiving and transmitting drought signals. These pathways are integrated with calcium and ROS-mediated signaling, facilitating rapid and coordinated stress responses. At the whole-plant level, root architectural traits, particularly those regulated by genes like *DRO1*, are consistently validated mechanisms for drought avoidance in field conditions by improving access to deep soil moisture. On the other hand, cellular protection mechanisms, such as LEA proteins and membrane stabilizers, contribute to stress tolerance but exhibit more context-dependent effects. These mechanisms operate within an interconnected regulatory hierarchy, suggesting that targeting key regulatory hubs and combining complementary traits offer greater potential than manipulating single genes in isolation. Future progress will rely on integrating multi-omics data with field-based phenotyping to prioritize genes with stable, cross-environment effects and expedite their incorporation into breeding programs for developing drought-resilient rice varieties.
