# Supplementary material for: Stress-tolerance genes in rice: bridging gene discovery, functional validation, and breeding applications
Source: Front Plant Sci. 2026 Jun 8;17:1835666. doi: 10.3389/fpls.2026.1835666 (PMC13288485; doi:10.3389/fpls.2026.1835666)
Supplement: Supplementary file 1 [file Table1.docx]

Table S1. Functionally validated genes

| **Gene** | **Validation Method** | **Drought Phenotype / Effect** | **Reference / Evidence** |
| --- | --- | --- | --- |
| *OsbZIP23* | Overexpression | Improved drought tolerance via ABA signaling | (Y. Yang et al., 2022) |
| *OsbZIP46* | Overexpression (active form) | Enhanced drought and osmotic tolerance | (Y. Yang et al., 2022) |
| *OsbZIP62* | OE & mutant | Improved drought tolerance; mutants more sensitive | (S. Yang et al., 2019) |
| *OsbZIP12* | Overexpression | Enhanced ABA sensitivity and drought tolerance | (Geng et al., 2024b) |
| *OsbZIP33* | Overexpression | Increased expression of drought genes | (Geng et al., 2024a) |
| *OsbZIP16* | Overexpression | Positive regulator of drought resilience | (Geng et al., 2024a) |
| *OsbZIP86* | Overexpression | Increased ABA content & drought tolerance | (Geng et al., 2024a) |
| *OsDREB1A* | Overexpression | Enhanced drought tolerance | (Geng et al., 2024a) |
| *OsDREB1B* | Overexpression | Improved drought resistance | (Geng et al., 2024a) |
| *OsDREB1F* | Overexpression | Positive drought response | (Todaka et al., 2015) |
| *OsDREB2A* | Overexpression | Increased drought tolerance | (Todaka et al., 2015) |
| *OsDREB2B* | Overexpression | Improved drought & heat tolerance | (Todaka et al., 2015) |
| *AP37 (AP2/ERF TF)* | Overexpression | Enhanced drought tolerance | (Todaka et al., 2015) |
| *OsERF3* | Overexpression | Altered drought response | (Todaka et al., 2015) |
| *OsERF83* | Overexpression/knockout | Increased drought tolerance | (Jung et al., 2021) |
| *SNAC1* | Overexpression | Reduced water loss, improved tolerance | (Todaka et al., 2015) |
| *OsNAC5* | Overexpression | Enhanced root traits & drought tolerance | (Todaka et al., 2015) |
| *OsNAC6* | Overexpression | Increased drought & salinity resilience | (Todaka et al., 2015) |
| *OsNAC9* | Overexpression | Improved drought tolerance & root diameter | (Bhattacharjee et al., 2023) |
| *OsNAC10* | Root‑specific OE | Thicker roots, improved drought yield | (Todaka et al., 2015) |
| *OsMYB2* | Overexpression | Enhanced tolerance to drought, salt, cold | (Todaka et al., 2015) |
| *ZFP182* | Overexpression | Enhanced drought & salt tolerance | (Todaka et al., 2015) |
| *ZFP252* | Overexpression | Increased proline & drought tolerance | ([Nature](https://www.nature.com/articles/s41598-023-45661-8?utm_source=chatgpt.com)) |
| *OsCPK4* | Overexpression | Membrane protection & drought tolerance | ([PMC](https://pmc.ncbi.nlm.nih.gov/articles/PMC4332304/?utm_source=chatgpt.com)) |
| *OsCDPK1* | Overexpression | Improved drought tolerance | ([PMC](https://pmc.ncbi.nlm.nih.gov/articles/PMC4332304/?utm_source=chatgpt.com)) |
| *GF14c* | Overexpression | Enhanced drought tolerance via signaling | ([PMC](https://pmc.ncbi.nlm.nih.gov/articles/PMC4332304/?utm_source=chatgpt.com)) |
| *OsCDPK7* | Overexpression | Enhanced drought & salinity tolerance | ([PMC](https://pmc.ncbi.nlm.nih.gov/articles/PMC4332304/?utm_source=chatgpt.com)) |
| *OsCIPK12* | Overexpression | Increased drought tolerance | ([PMC](https://pmc.ncbi.nlm.nih.gov/articles/PMC4332304/?utm_source=chatgpt.com)) |
| *OsSIK1* | Overexpression | Enhanced drought tolerance | ([PMC](https://pmc.ncbi.nlm.nih.gov/articles/PMC4332304/?utm_source=chatgpt.com)) |
| *OsSIK2* | Overexpression | Improved drought response | ([PMC](https://pmc.ncbi.nlm.nih.gov/articles/PMC4332304/?utm_source=chatgpt.com)) |
| *OsOAT* | Overexpression | Confers drought & oxidative stress tolerance | ([Notulae Botanicae](https://www.notulaebotanicae.ro/index.php/nbha/article/view/12128?utm_source=chatgpt.com)) |
| *OsSRO1c* | OE/Target of SNAC1 | Modulates stomatal closure & ROS tolerance | ([Notulae Botanicae](https://www.notulaebotanicae.ro/index.php/nbha/article/view/12128?utm_source=chatgpt.com)) |
| *OsPHYB* | Functional variant | Involved in drought root regulation | ([Notulae Botanicae](https://www.notulaebotanicae.ro/index.php/nbha/article/view/12128?utm_source=chatgpt.com)) |
| *OsERF71* | Overexpression | Increased drought resistance phenotype | ([Frontiers](https://www.frontiersin.org/journals/plant-science/articles/10.3389/fpls.2017.01044/full?utm_source=chatgpt.com)) |
| *OsWRKY11* | Overexpression | Enhanced drought tolerance | ([Nature](https://www.nature.com/articles/s41598-023-45661-8?utm_source=chatgpt.com)) |
| *OsWRKY114* | Overexpression | Negative regulation of drought tolerance | ([Nature](https://www.nature.com/articles/s41598-023-45661-8?utm_source=chatgpt.com)) |
| *OsmiR535* | Knockout | Increased drought tolerance | ([Springer](https://link.springer.com/article/10.1007/s40502-023-00743-7?utm_source=chatgpt.com)) |
| *OsCCR10* | Overexpression/knockout | Lignin mediated improved drought resistance | ([Springer](https://link.springer.com/article/10.1007/s40502-023-00743-7?utm_source=chatgpt.com)) |
| *OsERF65ZS97B* | Overexpression/KO | Modified drought response | ([ScienceDirect](https://www.sciencedirect.com/science/article/pii/S2667064X26000138?utm_source=chatgpt.com)) |
| *OsAAA‑1* | Knockout | Increased drought tolerance | ([Omics Online Publishing](https://www.omicsonline.org/open-access-pdfs/knockouts-of-drought-sensitive-genes-improve-rice-grain-yield-under-both-drought-and-wellwatered-field-conditions.pdf?utm_source=chatgpt.com)) |
| *OsAAA‑2* | Knockout | Increased drought tolerance | ([Omics Online Publishing](https://www.omicsonline.org/open-access-pdfs/knockouts-of-drought-sensitive-genes-improve-rice-grain-yield-under-both-drought-and-wellwatered-field-conditions.pdf?utm_source=chatgpt.com)) |
| *OsbZIP71* | Overexpression | Confers drought tolerance | ([Scholars Junction](https://scholarsjunction.msstate.edu/cgi/viewcontent.cgi?article=6256&context=td&utm_source=chatgpt.com)) |
| *PYL10* | Overexpression | Improved drought & cold tolerance | ([PMC](https://pmc.ncbi.nlm.nih.gov/articles/PMC11675997/?utm_source=chatgpt.com)) |
| *(Many Additional NAC, bZIP, DREB TFs)** | OE/KO | Various levels of drought tolerance | Reviews summary ([SpringerLink](https://thericejournal.springeropen.com/articles/10.1186/s12284-022-00614-z?utm_source=chatgpt.com)) |
